# Pharmacogenetic-Guided Algorithm to Improve Daily Dose of Warfarin in Elder Han-Chinese Population

**DOI:** 10.3389/fphar.2020.01014

**Published:** 2020-07-10

**Authors:** Yirong Ren, Chenguang Yang, Hao Chen, Dapeng Dai, Yan Wang, Huolan Zhu, Fang Wang

**Affiliations:** ^1^ Department of Cardiology, Beijing Hospital, National Center of Gerontology, Institute of Geriatric Medicine, Chinese Academy of Medical Sciences, Beijing, China; ^2^ Graduate School of Peking Union Medical College, Chinese Academy of Medical Science, Beijing, China

**Keywords:** atrial fibrillation, elder Han-Chinese, warfarin, genetic polymorphism, algorithm

## Abstract

**Objectives:**

To verify the accuracy of the International Warfarin Pharmacogenetics Consortium (IWPC) algorithm, identify the effects of genetic and clinical factors on warfarin stable dose, and to establish a new warfarin stable dose prediction algorithm for the elderly Han-Chinese population under the guidance of pharmacogenetics.

**Methods:**

According to the inclusion criteria, 544 non-valvular atrial fibrillation patients taking warfarin for anticoagulation treatment were enrolled. Data information of three groups including the whole population, people under 65 years old and over 65 years old were substituted into the IWPC algorithm respectively to verify its accuracy. The basic data and clinical information of 360 elderly people were collected for statistical analysis and the genotypes of VKORC1-G1639A and CYP2C9 were detected by Sanger sequencing. The new algorithm of the elder pharmacogenetics warfarin dosing was obtained by stepwise multiple regression. The determination coefficient (R2), root mean squared error (RMSE), and the proportion of the predicted value within the true value range of ±20%(20%-p) were used to evaluate the accuracy of the IWPC algorithm and the new algorithm.

**Results:**

Among the three different age groups, the warfarin stable dose predictive accuracy of IWPC algorithm was the lowest in the elderly patients above 65-year-old. In this study, the important factors influencing the stable dose of warfarin in the elderly Han-Chinese were height, weight, body surface area, serum creatinine level, amiodarone usage, *CYP2C9* (*1*2, *1*3), and *VKORC1* (GG/GA) genotypes. By means of stepwise multiple regression analysis, we established a new elder warfarin dosing algorithm (R^2^=0.3714) containing height, creatinine, amiodarone usage, *CYP2C9* (*1*2 or *1*3), and *VKORC1* (GA or GG) genotypes. The prediction accuracy and clinical availability of the Elderly algorithm was significantly better than that of IWPC algorithm verified by RMSE, R2, and (20%-p) methods.

**Conclusions:**

The IWPC model may not be suitable for the elder Han-Chinese population. Polymorphism of *CYP2C9* and *VKORC1* obviously affected warfarin stable dose of the elder Han-Chinese. Combination of genetic data with demographic and clinical factors could help to better improve warfarin doses in the elder Han-Chinese population.

## Introduction

Warfarin is the most commonly used anticoagulant for treatment and prophylaxis of thromboembolic diseases, such as pulmonary embolism and atrial fibrillation (Kimmel et al., 2013). However, the narrow therapeutic window and individual variation of warfarin dose make it difficult to achieve desired anticoagulation effect. The international normalized ratio (INR) is used to monitor the anticoagulation status as a standardized measurement and the most common target INR is 2.0 to 3.0. The higher INR than 3.0 could lead to a high risk of bleeding while the INR lower than 2.0 may result in a high risk of thrombosis (Johnson, 2012). A number of factors including non-genetic and genetic factors affect warfarin dose requirements. The clinical and environmental factors involved in the metabolism of warfarin are race, age, sex, body surface area (BSA), smoking, drug combination, renal insufficiency, hepatic disease, absorption or elimination of vitamin K (Dilge Taşkın et al., 2016; Takeuchi et al., 2016). The potential usage of genetic polymorphisms for the prediction of warfarin dose requirements was first recognized in 1999. Subsequently, numerous studies displayed that gene polymorphisms could influence warfarin dose requirements (Johnson, 2012). Especially, more and more evidences suggested that genetic variants of the cytochrome P450 complex subunit 2C9 (*CYP2C9*) and vitamin K epoxide reductase complex subunit 1 (*VKORC1*) greatly affected effective warfarin dose (Bourgeois et al., 2016; Farzamikia et al., 2017; Liu et al., 2019). Observational studies also demonstrated that age, body mass index, and polymorphism of *CYP2C9* and *VKORC1* accounted for nearly 50% of individual variations in warfarin stable dose (Bourgeois et al., 2016).

To date, more than 60 variant alleles in *CYP2C9* have been described and the most common allele of *CYP2C9* is ^⁎^1, usually considered as the wild-type genotype. *CYP2C9* ^⁎^2 and *CYP2C9* ^⁎^3 have been examined with respect to warfarin dosing (Flora et al., 2017; Johnson et al., 2017; Pirmohamed, 2018) and it is found that patients who have one or two copies of *CYP2C9* ^*^2 or ^*^3 require lower warfarin dose to achieve anticoagulation effect than those with *CYP2C9* ^*^1 homozygous and have a greater risk of bleeding during therapy (Johnson et al., 2011; Takeuchi et al., 2016; Cullell et al., 2018). Furthermore, warfarin is a specific inhibitor of the vitamin K epoxide reductase, encoded by the vitamin K epoxide reductase complex subunit 1 (*VKORC1*) gene. A common noncoding variant (*VKORC1*−1639G>A, rs9923231) has been shown definite association with warfarin sensitivity and dose requirements (Johnson et al., 2017). Patients with one or two -1639A alleles require lower warfarin doses than -1639G homozygotes (Wakamiya et al., 2016; Flora et al., 2017). Given all these findings, the US Food and Drug Administration revised warfarin product label in 2010 by including dose recommendations based on *CYP2C9* and *VKORC1* genotype (Johnson, 2012). The result of a recent-published randomized clinical trial further supported the fact that genetic variants affected warfarin dosage by showing that patients undergoing elective hip or knee arthroplasty and treated with perioperative warfarin in a genotype-guided dosing had lower risk of major bleeding, venous thromboembolism, and death (Gage et al., 2017).

In recent years, many dosing algorithms have been generated incorporating with both genetic and non-genetic factors in order to predict warfarin dose (Bourgeois et al., 2016; Li et al., 2016). The most classic pharmacogenetic warfarin dosing algorithm was the International Warfarin Pharmacogenetics Consortium (IWPC) algorithm, which substituted 5,000 patients from 4 continents into pharmacogenetic algorithm and could explain 46% interindividual variability (Johnson, 2012). But some studies have proved that IWPC algorithm was not suitable for the Han-Chinese (Peng et al., 2015). To our knowledge, few studies have been published on the pharmacogenetic-guided warfarin dosing algorithms in the elder Han-Chinese population so far. This study aimed to verify the accuracy of IWPC algorithm in different Chinese age groups and identified the effects of genetic and clinical factors on warfarin stable dose in the elderly Han-Chinese population, and at the same time established a new warfarin stable dose prediction algorithm for the elderly Han-Chinese population under the guidance of pharmacogenetics.

## Methods

### Ethics and Permissions

The investigation was approved by the Beijing Hospital Ethics Committee. And the written informed consent was obtained from all patients after discussion of the aim of the investigation..

### Inclusion Criteria

1. Han-Chinese population, ≥18 years old;
2. Patients with non-valvular atrial fibrillation;
3. Receive warfarin anticoagulant therapy for at least 3 months, INR reaches target range 2.0–3.0;
4. Not simultaneously taking drugs that affect the metabolism of warfarin (except amiodarone and statins);
5. Understand the study in detail and sign the informed consent.

### Data Collection

The study included 544 Han-Chinese patients with nonvalvular atrial fibrillation who received warfarin therapy at Beijing Hospital, Tongren Hospital, Xuanwu Hospital, Anzhen Hospital, and Tiantan Hospital. Three groups were classified including the whole population group with the overall 544 patients, the elderly group of 360 patients over 65 years old and the non-elderly group with the remaining 184 patients under 65 years old. The accuracy of IWPC algorithm was verified by the above three groups respectively. We recorded clinical and genetic data of all patients between January 2016 and May 2019. The clinical information included age, gender, height, weight, body surface area (BSA), smoke, serum creatinine, concomitant drugs such as amiodarone and statins. The anticoagulation data included INR values and warfarin stable dose. The warfarin stable dose, demographic and clinical information were collected in patients with stable INR of 2.0–3.0. The warfarin stable dose was defined as the average daily dose of warfarin (mg/day, weekly dose/7 days) when the INR was in the target range (2.0 to 3.0) for at least 2 consecutives (at least 7 to 14 days) days after warfarin treatment.

### DNA Extraction and Genotyping

Genomic DNA was extracted from 2 ml peripheral blood and standardized to approximately 100 ng/µl for polymerase chain reaction (PCR). Exons of CYP2C9 and promoter region of VKORC1 were amplified and sequenced using modified primers based on the previous study (Dai et al., 2014). Detailed information for these primers was provided in supplementary materials. The amplified products were purified using the gel purification system (Omega Bio-Tek Inc, Norcross, GA, USA) and sequenced using the ABI Prism Big Dye Terminator Cycler Sequencing Kit (Applied Biosystems, Foster City, CA, USA) on the ABI 3730xl DNA Analyzer. All the sequences were verified by at least two individuals and bidirectional sequencing was performed for the putatively mutated sites.

### Statistical Analysis

SPSS 21.0 software was used for statistical analysis. Graphics were generated with the ggplot2 R (version 3.5.2) software package. The measurement data were statistically described using X ± SD. For the data conforming to normal distribution, two independent samples t-test was used to compare the differences between groups, and Pearson correlation analysis was used to test the correlation between the two variables. Categorical data were compared between groups using χ^2^ test and variance analysis. The correlation between the warfarin stable dose and different variables was analyzed by the above statistical methods in order to screen the variables closely related to warfarin stable dose. *P* < 0.05 was considered statistically significant and *P* < 0.01 was considered very statistically significant. The data of different populations were substituted into the IWPC algorithm to verify accuracy. A new dose prediction algorithm was obtained by integrating the related variables into stepwise multiple linear regression. The trend correlation between the predicted value and the true value was evaluated by the determination coefficient (R^2^). The root mean squared error (RMSE) was used to evaluate the algorithm fitting and prediction accuracy and the value of RMSE was negatively correlated with prediction accuracy. The proportion of the predicted value within the true value range of ±20% (20%-p) was used to evaluate the clinical availability of the algorithm. The value of 20%-p was positively correlated with clinical availability. All single nucleotide polymorphisms were tested by χ^2^ test for deviations from the Hardy–Weinberg equilibrium.

## Results

### IWPC Algorithm Validation

**Figure 1:**
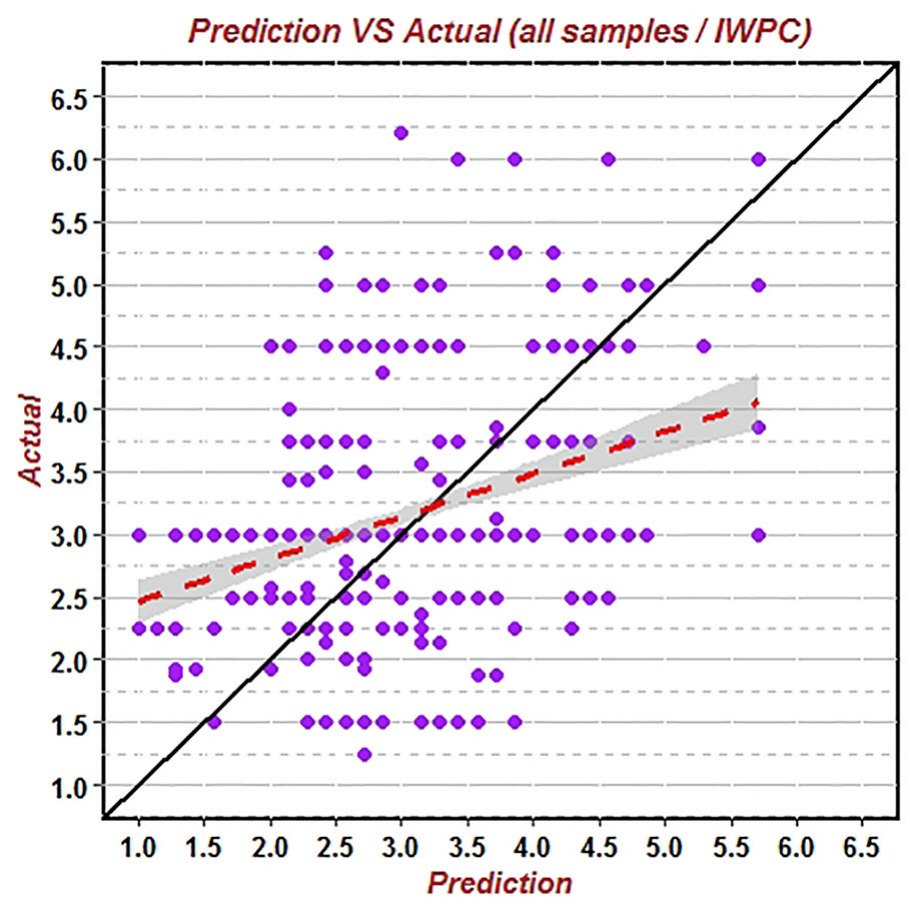
Predicted vs. actual warfarin stable dose verified by IWPC algorithm using data of the 544 whole patients.

**Figure 2:**
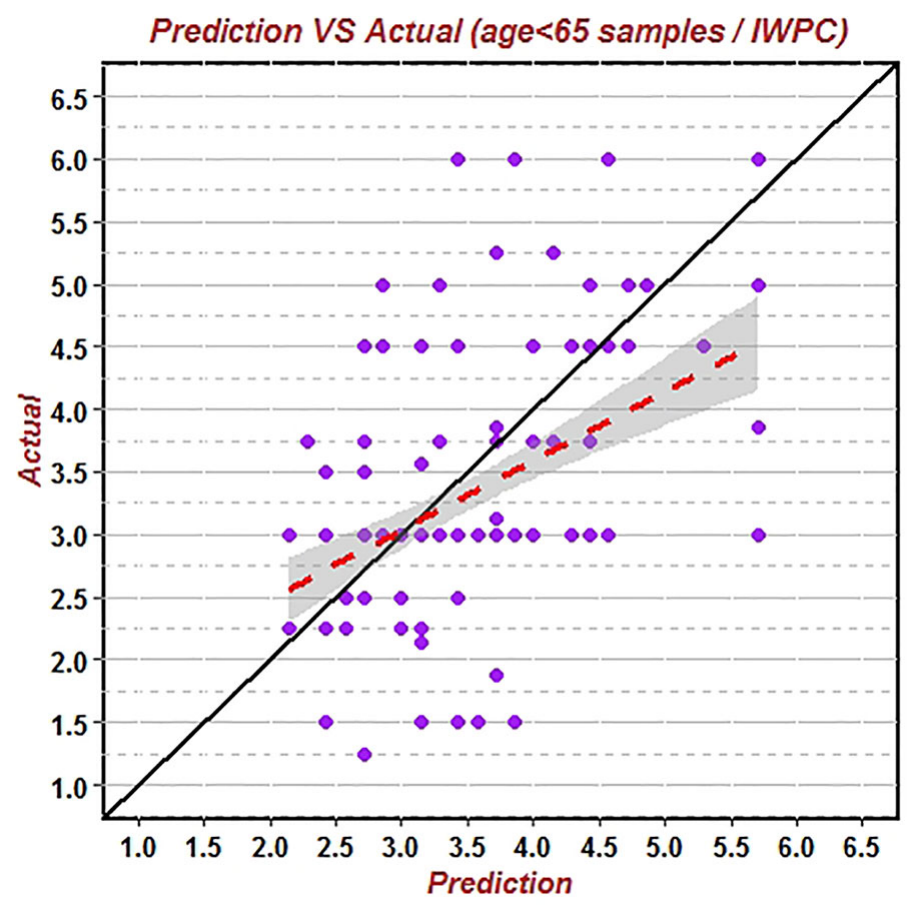
Predicted vs. actual warfarin stable dose verified by IWPC algorithm using data of patients under 65 years old.

**Figure 3:**
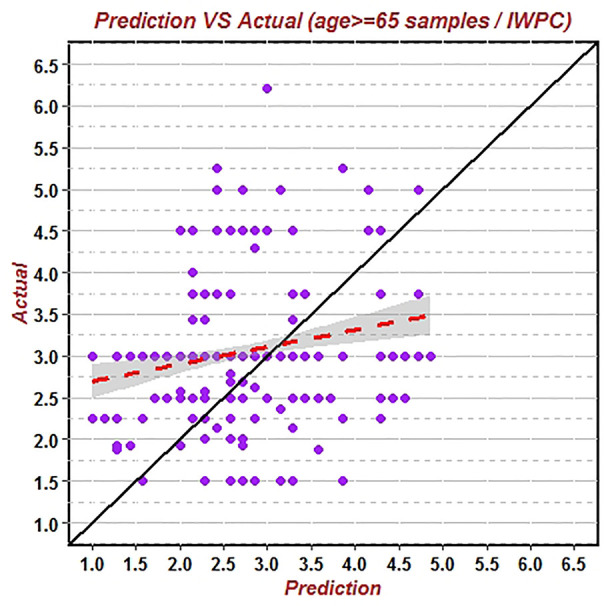
Predicted vs. actual warfarin stable dose verified by IWPC algorithm using data of patients above 65 years old.

First, the data of the whole population group (544 patients) was substituted into the IWPC algorithm to verify its prediction accuracy of warfarin stable dose. The results showed that the correlation coefficient (R^2^) of the predicted value and the true value was 0.1321, RMSE was 1.0137, and the proportion of the predicted value within the true value ±20% (20%-p) was 55.15% (**Figure 1**). Similarly, the data of the non-elderly group (184 patients) and the elderly group (360 patients) were substituted into the IWPC algorithm respectively. The results showed that R^2^ of the non-elderly group was 0.2293, RMSE was 0.9376 and (20%-p) was 61.75% (**Figure 2**). In the elderly group, the correlation coefficient R^2^, RMSE and (20%-p) verified by the IWPC algorithm were 0.0822, 1.0502, and 51.80% respectively (**Figure 3**). Compared with the whole population group and the non-elderly group, we found that R^2^ and 20%-p of the elderly group were the lowest and RMSE was the largest. These results indicated that IWPC algorithm had the lowest prediction accuracy and clinical availability in the elderly group, which suggested that IWPC algorithm might not be suitable for the elder Han-Chinese population.

### Clinical and Demographic Factors

The study consisted of 360 patients older than 65 years, including 232 males (64.44%) and 128 females (35.56%). Patients’ characteristics were shown in **Table 1**. The mean age, weight, height, and BSA were 74.08 ± 6.30 years, 71.04 ± 12.38 kg, 167.50 ± 8.65 cm, and 1.78 ± 0.19 kg/m^2^ respectively. Mean serum creatinine was 79.71 ± 17.31 µmol/L. Among them, 59 patients (16.39%) took amiodarone and 85 patients (23.61%) took statins as concomitant medication with warfarin. The mean warfarin dosage was 3.10 ± 0.91 mg/d. It was found that four factors, including weight, height, body surface area, and creatinine, had significant effects on the stable dose of warfarin (*P* = 0.007; *P* < 0.001; *P* < 0.001; *P* = 0.045) (**Tables 2** and **3**).

**Table 1:** Baseline data analysis of people over 65 years old.

Factors	N (%) or mean ± SD
Age, y Male patients/female patients	74.08 ± 6.30 232 (64.44)/128 (35.56)
Smoking/not	50 (13.89)/310 (86.11)
Amiodarone/not	59 (16.39)/301 (83.61)
Statins/not	85 (23.61)/275 (76.39)
Height, cm	167.50 ± 8.65
Weight, kg	71.04 ± 12.38
BSA, m^2^	1.78 ± 0.19
Creatinine, µmol/L	79.71 ± 17.31
Warfarin dosage, mg/d	3.10 ± 0.91

BSA, body surface area; Quantitative traits are presented as mean ± SD.

**Table 2:** Comparison of stable warfarin dose in elder patients with clinical factors.

Factors	Classification	Stable dose of warfarin (mg/d)	*P*
Sex	Male(232)	3.19 ± 1.01	0.085
	Female(128)	2.94 ± 0.68	
Age, y	65-74	3.14 ± 0.95	0.385
	≥75	3.05 ± 0.87	
Smoking	yes	3.26 ± 1.33	0.927
	no	3.08 ± 0.82	
Amiodarone	yes	2.93 ± 0.77	0.103
	no	3.14 ± 0.93	
Statins	yes	2.99 ± 0.90	0.209
	no	3.14 ± 0.91	
Creatinine (µmol/L)	≥130	2.14 ± 0.77	0.045
	<130	3.11 ± 0.91	

Stable dose of warfarin are presented as mean±SD. P < 0.05 was considered statistically significant.

**Table 3:** Pearson correlation analysis between height, weight, BSA with warfarin stable dose.

	r	R^2^	*t*	*P*
Weight (kg)	0.141	0.058	2.627	0.007
Height (cm)	0.251	0.063	4.747	<0.001
BSA (m^2^)	0.184	0.034	3.448	<0.001

BSA, body surface area; P < 0.05 was considered statistically significant.

### SNP Analysis

Genotype frequencies for *VKORC1*-1639 G>A and *CYP2C9* in the study population older than 65 years were shown in **Table 4**. Three *CYP2C9* alleles were detected: *CYP2C9* ^*^1, *CYP2C9* ^*^2, and *CYP2C9* ^*^3, respectively with allelic frequencies of 96.39%, 0.14%, and 3.47%. *CYP2C9* ^*^1^*^1 genotype as wild type was most common with a frequency of 92.78%, followed by ^*^1^*^3 genotype of 6.94% and the rare ^*^1^*^2 genotype accounted for 0.28%. Three genotypes AA, GA, and GG were detected in *VKORC1*-1639G>A polymorphism with the frequency of 83.06%, 16.11%, and 0.83% respectively. All single nucleotide polymorphisms were tested for deviations from the Hardy–Weinberg equilibrium (*P* > 0.05).

**Table 4:** Distribution of *CYP2C9* and *VKORC1* genotypes.

	genotype	N (%)
*CYP2C9*	^*^1^*^1	334(92.78)
	^*^1^*^3	25(6.94)
	^*^1^*^2	1(0.28)
*VKORC1*-1639G>A	AA	299(83.06)
	GA	58(16.11)
	GG	3(0.83)

### Genotype and Warfarin Dose

**Figure 4:**
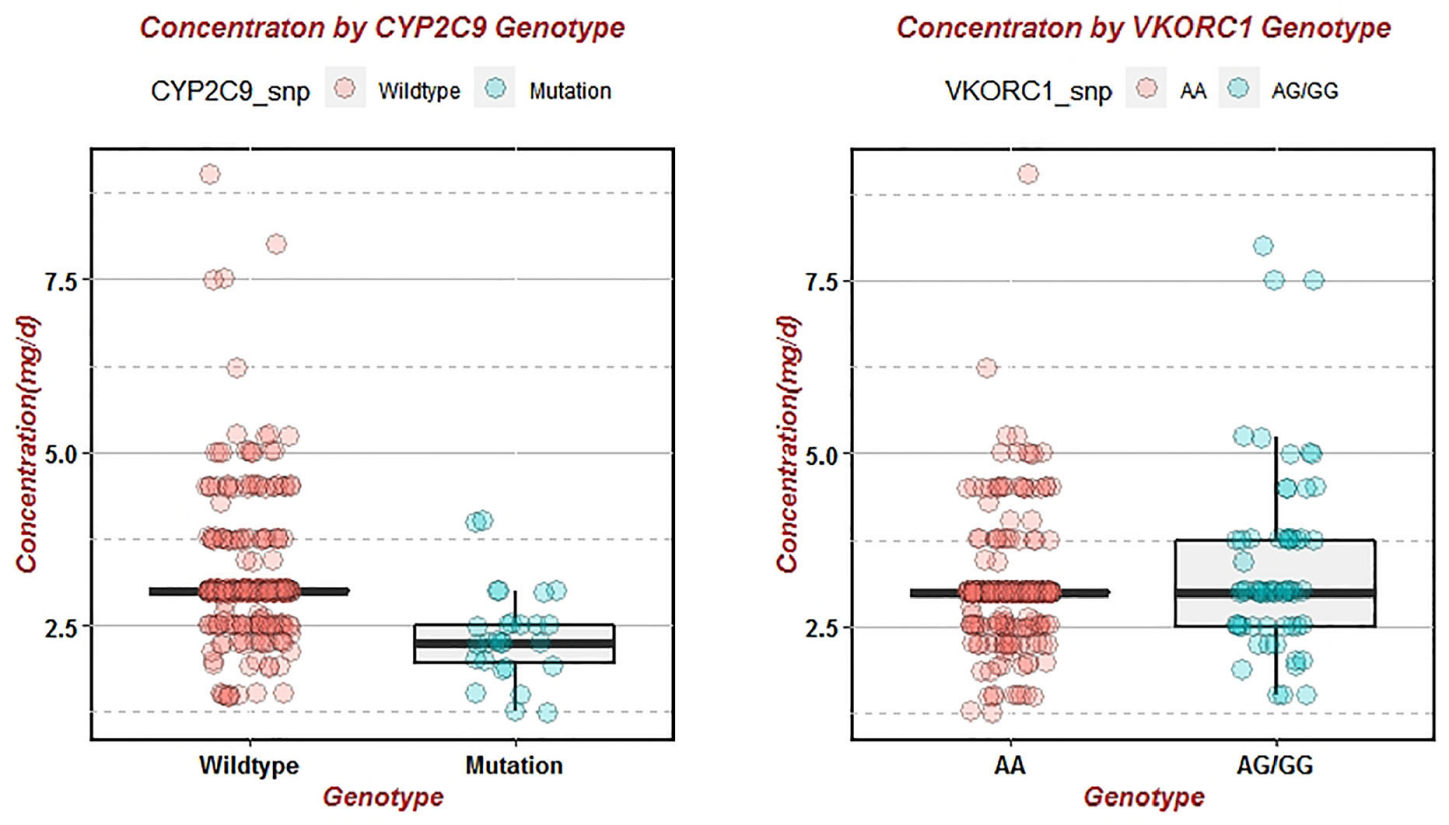
Effects of wild-type and mutant of *CYP2C9* and *VKORC1* on warfarin stable dose. Variance analysis of warfarin dose in different genotypes was represented by boxplot, and scatter was the true warfarin dose distribution of each patient with different genotypes.

The warfarin stable dose in patients with the wild-type *CYP2C9* ^*^1^*^1 was significantly higher than those with the heterozygote (3.16 ± 0.90 mg/d vs. 2.39 ± 0.67 mg/d, *P* < 0.001) (**Table 5**). Patients with a mutant homozygotic AA genotype of *VKORC1* -1639G>A required lower maintenance dose than those with the heterozygotic GA and GG genotype [3.0(3.0,3.0) vs. 3.0(2.5,3.75) mg/d, *P* < 0.046]. (**Table 5**). There was a statistical significance in the warfarin stable dose between the patients with wildtype and mutant of *CYP2C9* (**Figure 4**). Patients with *VKORC1* AA versus GG/GA were also demonstrated significantly different in view of the warfarin stable dose (**Figure 4**). *CYP2C9* wild-type ^*^1^*^1 and *VKORC1* (AA) together were the most common genotypes (275 cases, 76.39%), while ^*^1^*^1 combined with GA or GG were detected in 91 patients (16.39%) with the highest dose of warfarin treatment [3.0(2.5,3.75) mg/d, *P* < 0.001] (**Table 6**). In patients with *CYP2C9* mutant and *VKORC1* GA/GG combination, the stable dose of warfarin was 2.0(2.0,2.0) mg/d. But only two patients carried the combination of *CYP2C9* (*1*3/*1*2) and *VKORC1* (GA/GG) genotypes, which made it difficult to compare the stable dose of warfarin with other combinations accurately (**Table 6**). The results of correlation test showed that *CYP2C9* (*1*3, *1*2) was negatively correlated with warfarin dose (*r* = -0.219, *R* ^2^= 0.0480, *P* < 0.001) while the *VKORC1* (GA, GG) genotype was positively correlated with dose (*r* = 0.139, *R* ^2^= 0.0193, *P* = 0.03) (**Table 7**).

**Table 5:** Comparison of warfarin stable dose in patients with different genotypes.

Gene	Warfarin dose (mg/d)	P
***CYP2C9***		<0.001
*1*1	3.16±0.90	
*1*3, *1*2	2.39±0.67	
***VKORC1***		0.046
AA	3.0(3.0,3.0)	
GA, GG	3.0(2.5,3.75)	

The warfarin stable dose in different VKORC1 genotypes are expressed as median (interquartile range). The stable dose of patients with different CYP2C9 genotypes are expressed as mean±SD. P < 0.05 was considered statistically significant.

**Table 6:** Comparison of warfarin stable dose in patients with different genotypes combination.

CYP2C9	VKORC1	N(%)	Warfarin dose (mg/d)	P
^*^1^*^1	AA	275(76.39)	3.0(3.0,3.0)	<0.001
	GA, GG	59(16.39)	3.0(2.5,3.75)	
^*^1^*^2, ^*^1^*^3	AA	24(6.67)	2.38(2.01,2.88)	
	GA, GG	2(0.56)	2.0(2.0,2.0)	

The warfarin stable dose in different genotype combination are expressed as median (interquartile range). P < 0.05 was considered statistically significant.

**Table 7:** Pearson correlation analysis between genotypes and warfarin dose.

Genotype	*r*	*R* ^2^	*t*	*P*
*CYP2C9*(*1*2,*1*3)	-0.219	0.0480	-4.502	<0.001
*VKORC1* (GG, GA)	0.139	0.0193	2.691	0.008

R^2^, determination coefficient; P < 0.05 was considered statistically significant.

### Multiple Regression Algorithm

After random sampling, 240 patients were obtained for the stepwise multivariate regression to construct a new elder warfarin pharmacogenetic algorithm, and the remaining 120 patients were used for algorithm verification. Height (*P* < 0.001), creatinine (*P* = 0.0267), amiodarone usage (*P* < 0.001), *CYP2C9* (*1*2,*1*3)(*P* < 0.001), and *VKORC1* (GA, GG) (*P* = 0.0034) were incorporated into the multivariate regression algorithm (**Table 8**). The new algorithm was named as the Elderly algorithm (R^2^ = 0.3714, *P* < 0.001):

Y(mg/d)=10^[0.4578+0.2279×Height−0.1372×Creatinine−0.0862×Amiodarone−0.1426×CYP2C9(*1*2,*1*3)+0.0659*VKORC1(GA,GG)]

**Table 8:** Multivariate regression algorithm of population over 65 years.

Factors	*β*	*SE*	*t*	*P*
Constant term	0.4578	0.0338	13.524	<0.001
Height	0.2279	0.0564	4.041	<0.001
Creatinine	-0.1372	0.0611	-2.245	0.0267
Amiodarone	-0.0862	0.0250	-3.444	<0.001
*CYP2C9* (*1*2,*1*3)	-0.1426	0.0274	-5.191	<0.001
*VKORC1*(GG, GA)	0.0659	0.0220	2.991	0.0034

β is the coefficient in front of the independent factors; SE, standard error; P < 0.05 was considered statistically significant.

### Comparison of Verification Accuracy Between the Elderly Algorithm and IWPC Algorithm in Different Populations

**Figure 5:**
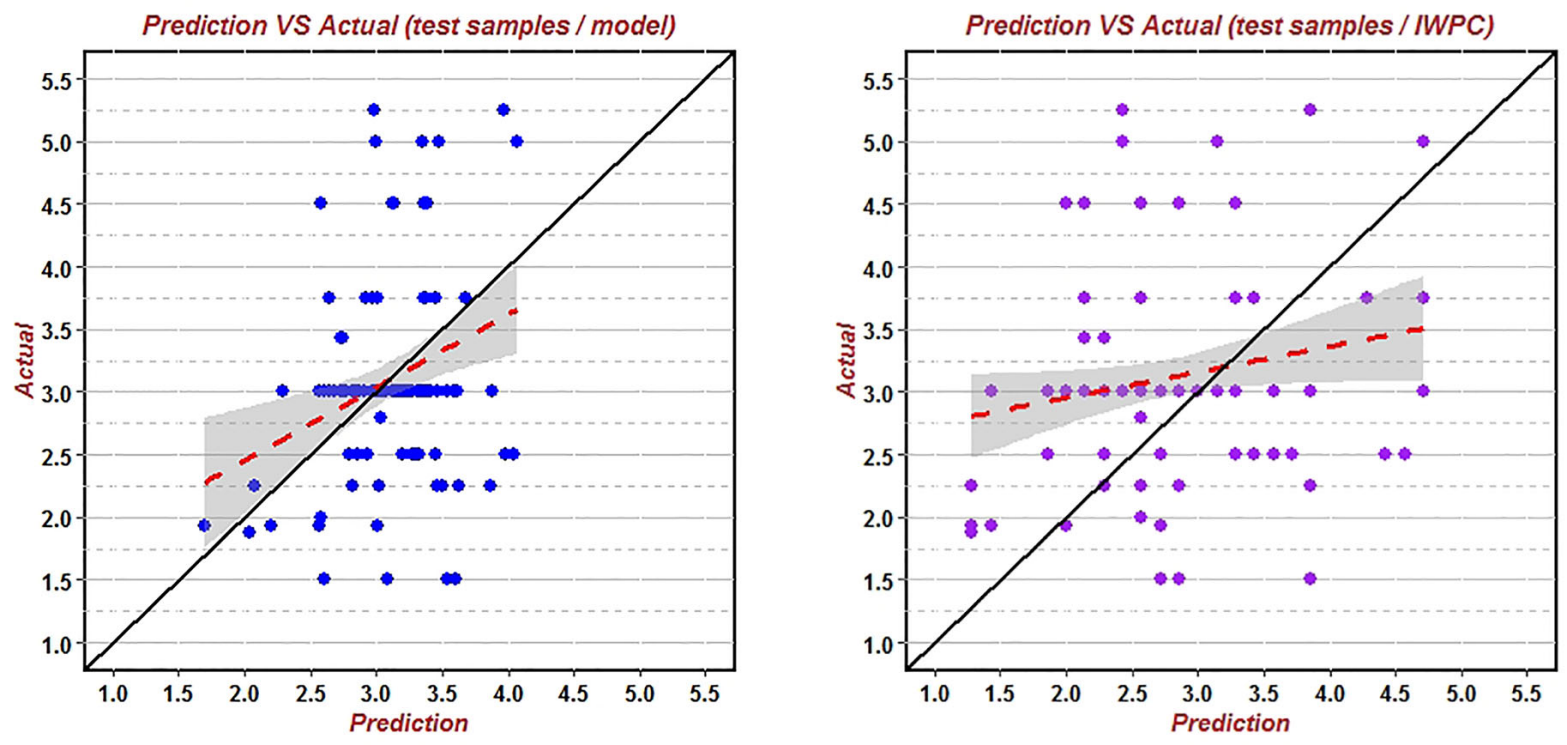
Predicted vs. actual warfarin stable dose verified by the new algorithm and IWPC algorithm using data of the remaining 120 patients over 65years. This was a scatter plot of the correlation between the predicted value and the actual value. The red line was the fitting line, and the black line was the theoretical line where the actual value and the predicted value were equal.

The remaining 120 patients of the validation group were substituted into the new elder algorithm to verify accuracy (**Figure 5**). The predicted value of warfarin dose was 1.796 mg/d (95% CI: 1.129–2.857) to 4.434 mg/d (95% CI: 2.796–7.031). The correlation coefficient R2 was 0.0125, RMSE was 0.9937, and the proportion of the predicted value within the true value ±20% (20%-p) was 59.50% (**Table 9**). Meanwhile, the data of these 120 patients were also substituted into the classic IWPC algorithm (**Figure 5**), and the R2 was 0.0021, RMSE was 1.2297, and the (20%-p) was 45.45% (**Table 9**).

**Table 9:** Comparison of the two algorithms in different populations.

Patients Groups	N	The Elderly algorithm			IWPC algorithm		
		R^2^	RMSE	20%-p	R^2^	RMSE	20%-p
120 cases of the validation group	120	0.0125	0.9937	59.50%	0.0021	1.2297	45.45%
the whole population	544	0.1925	0.8613	66.91%	0.1321	1.0137	55.15%
all patients above 65-year-old	360	0.1510	0.8523	66.20%	0.0822	1.0502	51.80%

R^2^, determination coefficient; RMSE, root mean squared error; (20%-p), the proportion of the predicted value within the true value range.

It was found that compared with IWPC algorithm, the R^2^ and (20%-p) of the Elderly algorithm were large and RMSE was small (**Table 9**), which indicated that the Elderly algorithm was better than IWPC algorithm in predicting stable dose of warfarin in the elderly Han-Chinese population with greater accuracy and better clinical availability.

## Discussion

For decades, numerous studies have demonstrated the effects of *VKORC1* and *CYP2C9* gene polymorphism and clinical parameters on warfarin stable concentration, but few have considered the special group older than 65 years. In the patients older than 65 years, warfarin was one of the drugs responsible for a third of US emergency room visits with serious adverse reactions to medications (Budnitz et al., 2007). In our study, the effects of *VKORC1* and *CYP2C9* genetic variations on warfarin dose were investigated in the elder Han-Chinese population with nonvalvular atrial fibrillation. We also constructed a more suitable multivariate regression algorithm especially for those over 65 years.

The significance of the functional promoter polymorphism, *VKORC1*–1639G>A gene on the warfarin dose requirements has been studied by many researches (Zhang et al., 2015; Topkara et al., 2016; Li et al., 2018). A meta-analysis including 53 studies showed that most common *VKORC1* genotypes in Asian and Caucasian were -1639 AA, -1173 TT and -3730 GG, but the distribution frequency of these three genotypes in Asian was higher than that in Caucasian (Tang et al., 2017). About 90% of the Chinese population carried *VKORC1* variant allele whereas only 20% of the Caucasian carried these variations (Natarajan et al., 2013; Liu et al., 2019). Consistently in our study, the frequency of *VKORC1* variant allele was 91.11%. A recent meta-analysis have revealed that *VKORC1*-1639 GG, GA,and G carriers required a 101% (53.0%–149.0%), 40% (36.0%–45.0%) and 38% (35.0%–42.0%) higher mean daily warfarin dosage (MDWD), respectively than *VKORC1*-1639 AA carriers (Zhang et al., 2016). Our results were also consistent with their findings, elder patients who were *VKORC1* GG and GA genotype, required higher standard warfarin dose (3.38±1.35 mg/d vs. 3.05±0.78 mg/d, *P* < 0.001) to maintain target INR in the therapeutic range. There was a statistically significant difference in dose between the wild-type and mutant genotypes (*P* < 0.001) (**Tables 5** and **6**), which further confirmed the role of *CYP2C9* and *VKORC1* polymorphism in warfarin individual variability.

Multiple in vivo studies and clinical case reports have showed that the expression of *CYP2C9* ^*^2 and ^*^3 alleles significantly reduced the metabolism and daily dose requirements of selected *CYP2C9* substrates such as warfarin (Farzamikia et al., 2017; Flora et al., 2017). In particular, *CYP2C9* ^*^2 was virtually absent in Asians (Johnson et al., 2011). Among the 360 elder patients in this study, only one patient was found to carry the mutation type of ^*^2, which was parallel to its distribution pattern in the Asians (Yang et al., 2013). Previous studies have confirmed that individuals carrying one or two *CYP2C9* mutant alleles required lower dose of warfarin to reach the target INR value, but may be at higher risk of coagulation and bleeding, especially in the induction stage of treatment (Garcia et al., 2010; Yang et al., 2013). In accordance with our study, patients with *CYP2C9* wild-type ^*^1^*^1 required the highest warfarin stable dose than those with mutant genotypes (3.16 ± 0.90 mg/d. vs. 2.39 ± 0.67 mg/d, *P* < 0.001).

Genotype-guided warfarin dosing has been shown in numerous randomized trials to improve anticoagulation outcomes in individuals of European ancestry, yet its utility in Asian patients remains unresolved (Syn et al., 2018). In recent years, a lot of studies have developed different warfarin dose algorithms with both clinical factors and genetic polymorphisms (Schwartz et al., 2011; Natarajan et al., 2013; Wakamiya et al., 2016). However, there are scarce studies descripted gene-guided warfarin stable dose algorithms for the elderly over 65 years. A report of gene-directed warfarin dosing algorithm in the very elderly/frail elderly, enrolled only Caucasians and the algorithm could only explained 26.6% overall interindividual variability of the warfarin dose (Pautas et al., 2010). In our study, predictive accuracy of the warfarin stable dose by IWPC algorithm demonstrated to be weakest in the patients above 65-year-old among the three different age groups (**Figures 3** and **4**), which suggested that IWPC algorithm was not suitable for Han-Chinese elder patients.

In our study, height (P < 0.001), creatinine (P = 0.0267), amiodarone usage (P < 0.001), *CYP2C9* (*1*2,*1*3) (P < 0.001), and *VKORC1* (GA, GG) genotypes (P = 0.0034) were found to be the factors that most strongly influenced warfarin dosage in the population over 65 years. We established a new warfarin dosing algorithm based on these factors via multivariate regression (R^2^ = 0.3714). The data of 120 patients in the verification group were substituted into the new algorithm and the IWPC algorithm respectively. It was found that compared with IWPC algorithm, new algorithm had large correlation coefficient R^2^ (1.25 vs 0.21%), small root mean square error (0.9937 vs 1.2297) and large proportion of the predicted value within the true value ±20% (59.50 vs 45.45%). The comprehensive evaluation results showed that the population verification accuracy and clinical availability of the Elderly algorithm was significantly better than that of the IWPC algorithm, indicating that the new algorithm was more suitable for the elderly population of Han-Chinese to some extent. Considering that the classic IWPC algorithm was widely based on 5,000 patients, our new elder algorithm has better specificity and representativeness for the Han-Chinese elder population.

Our study has several limitations. We didn’t detect any new gene alleles such as ^*^36, ^*^39, ^*^46, and ^*^55 that could reduce *CYP2C9* metabolic activity. Complications of improper administration of warfarin such as bleeding or embolization were not recorded. The R^2^ of the Elderly algorithm was not very satisfactory possibly because of the small sample sizes, which indicated that we need large-size samples to optimize the algorithms and could incorporate other genes. Prospective trials are essential to validate the new warfarin algorithms in order to produce the best clinical results with warfarin.

## Conclusion

In summary, IWPC model may not be suitable for the elder Han-Chinese population. Both genetic and clinical factors affected warfarin stable daily dose in the elder Han-Chinese population. The new Elderly algorithm combining genetic data with demographic and clinical factors could help to better improve warfarin usage in the elder Han-Chinese population.

## Data Availability Statement

Patients’ data for this study were not publicly available for the time being because the study project has not been terminated. Requests to access these datasets should be directed to thankyourenyirong@163.com.

## Ethics Statement

The studies involving human participants were reviewed and approved by Beijing Hospital Ethics Committee. The patients/participants provided their written informed consent to participate in this study.

## Author Contributions

YR and FW designed the experiment. YR, CY, HC, and DD performed the experiment. YR and YW processed the data. YR wrote the paper. HZ and FW modified the paper.

## Funding

Our study was supported by the 135 National Science and Technology Major New Drug Creation Project (grant number: 2017ZX09304026) and the Capital Health Research and Development of Special (grant number: BJ-2016-071).

## Conflict of Interest

The authors declare that the research was conducted in the absence of any commercial or financial relationships that could be construed as a potential conflict of interest.

## Abbreviations

AF, Atrial Fibrillation; IWPC, International Warfarin Pharmacogenetics Consortium; INR, International Normalized Ratio; CYP2C9, CytochromeP450 Complex Subunit2C9; VKORC1, Vitamin K Epoxide Reductase Complex Subunit 1; CI, Conﬁdence Interval; BSA, body surface area; PCR, polymerase chain reaction; MDWD, Mean Daily Warfarin Dose.
